# Improved Sets of Artificial Teeth

**Published:** 1854-07

**Authors:** Mahlon Loomis

**Affiliations:** Cambridgeport, Mass.


### ARTICLE X.

*Improved Sets of Artificial Teeth.* By MAHLON LOOMIS, Cambridgeport, Mass.

MESSRS. EDITORS.—I have made an improvement in sets of artificial teeth which must revolutionize, to a great extent, that kind of business, and will be of much interest to every dentist, as well as to the community generally.

It is an improvement which I have just patented in this country, Great Britain and France, and consists chiefly in making whole or half sets of artificial teeth all of porcelain, without the use of any metallic plate, and a half set to consist of but one piece of material.

Many advantages arising from this, immediately suggest themselves. In the first place, a set made in this way will be a *neater* or more perfect manufacture than can be made in any other way; they cannot become impure—even with the utmost negligence they will keep themselves perfectly clean. Again, the expense of making them in this way is so comparatively small, that they may be afforded at a greater profit, at one half the price of sets made in the usual way. The labor and time required for making a set of teeth in this way is incomparably less, and dispenses at once with a multiplicity of tedious operations. I have fitted the most difficult gums with all the ordinary ease and accuracy, and several sets which I have made of this style have now been in use a year, answering every requirement, besides possessing many other self evident advantages.

I know from experience, that no dentist will ever go back to other methods after he has once made use of this. And *no* person who wears artificial teeth will ever wear any others when they have tried or seen these; for they have but to be seen and they evince their own superiority. That this way of making sets of teeth has long since been thought of, there is little doubt, but its practical availability is now made manifest.

